# Emerging Roles of *Circ-ZNF609* in Multiple Human Diseases

**DOI:** 10.3389/fgene.2022.837343

**Published:** 2022-07-22

**Authors:** Songbo Wang, Jiajin Wu, Zhongyuan Wang, Zixuan Gong, Yiyang Liu, Zengjun Wang

**Affiliations:** Department of Urology, First Affiliated Hospital of Nanjing Medical University, Nanjing, China

**Keywords:** circular RNA, ZNF609, human diseases, tumor malignant progression, mechanism

## Abstract

Circular RNAs (circRNAs) are a special type of endogenous RNAs with extensive roles in multiple human diseases. They are formed by back-splicing of partial sequences of the parental precursor mRNAs. Unlike linear RNAs, their covalently closed loop structure without a 5′ cap and a 3′ polyadenylated tail confers on them high stability and they are difficult to be digested by RNase R. Increasing evidence has proved that aberrant expressions of many circRNAs are detected and that circRNAs exert essential biological functions in disease development and progression *via* acting as a molecular sponge of microRNA, interacting with proteins as decoys or scaffolds, or self-encoding small peptides. Circular RNA zinc finger protein 609 (*circ-ZNF609*) originates from exon2 of ZNF609, which is located at chromosome 15q22.31, and it has recently been proved that it can translate into a protein. Being aberrantly upregulated in various diseases, it could promote malignant progression of human tumors, as well as tumor cell proliferation, migration, and invasion. Here in this review, we concluded the biological functions and potential mechanisms of *circ-ZNF609* in multiple diseases, which could be further explored as a targetable molecule in future accurate diagnosis and prognosis.

## Introduction

In 1976, circular RNAs (circRNAs) were found in viroids and eukaryotic cells (Kolakofsky, 1976; Sanger et al., 1976). In the process of transcription from parental gene to RNA, circular RNAs are back-spliced to form a loop structure without a 5′ cap and a 3′ polyadenylated tail (Jeck et al., 2012; Chen and Yang, 2015). The special circular structure endows them with a characteristic so that they can resist the digestion of exonuclease RNase R and become a class of relatively stable RNAs (Suzuki et al., 2006; Xiao and Wilusz, 2019).

With the development of high-throughput RNA sequencing and bioinformatics, the mechanisms and functions of circRNAs have been gradually elucidated (Memczak et al., 2013; Chen and Yang, 2015). According to the current research, circRNAs are generally divided into three subtypes: exonic circRNAs (ecircRNAs) (Jeck et al., 2012), exon–intron circRNAs (eicircRNAs) (Li et al., 2015), and intronic circRNAs (ciRNAs) (Zhang et al., 2013). The majority is ecircRNAs located in the cytoplasm mostly, and the other two exist in the nucleus mostly (Jeck et al., 2012; Zhang et al., 2013; Li et al., 2015). CircRNAs are ubiquitous in tissue cells, blood cells, serum, and exosomes (Salzman et al., 2012; Rong et al., 2019; Shi et al., 2020a). The primary functions of circRNAs are as follows: the ability to regulate the transcription of parental mRNAs (Li et al., 2015); acting as molecular sponges to regulate the expressions of target genes (Thomson and Dinger, 2016); binding to and sequestering proteins to regulate the expressions of the associated proteins (Kristensen et al., 2019; Chen, 2020); and translating into proteins to perform their functions (Legnini et al., 2017; Shi et al., 2020b). CircRNAs play important roles in the occurrence and development of human diseases, such as promoting cell proliferation, migration, and invasion in tumors, regulating drug resistance, and the progression of cardiovascular disease, as well as regulating neovascularization (Liu et al., 2017; Aufiero et al., 2019; Rossi et al., 2019; Li et al., 2020a; Xiao et al., 2020; Ding et al., 2021; Kim et al., 2021; Verduci et al., 2021).

Circular RNA zinc finger protein 609 (*circ-ZNF609*) is highly expressed in normal human neurons and maintains physiological functions (Rybak-Wolf et al., 2015; Legnini et al., 2017). It is differentially expressed in a variety of human diseases, as a competitive endogenous RNA to regulate target genes and affect disease progression. In the present review, we aim to gain insights into the relationship between *circ-ZNF609* and human diseases and provide a theoretical basis for clinical diagnosis and targeted therapy.

### Structure and Biological Function of *Circ-ZNF609*

**FIGURE 1:**
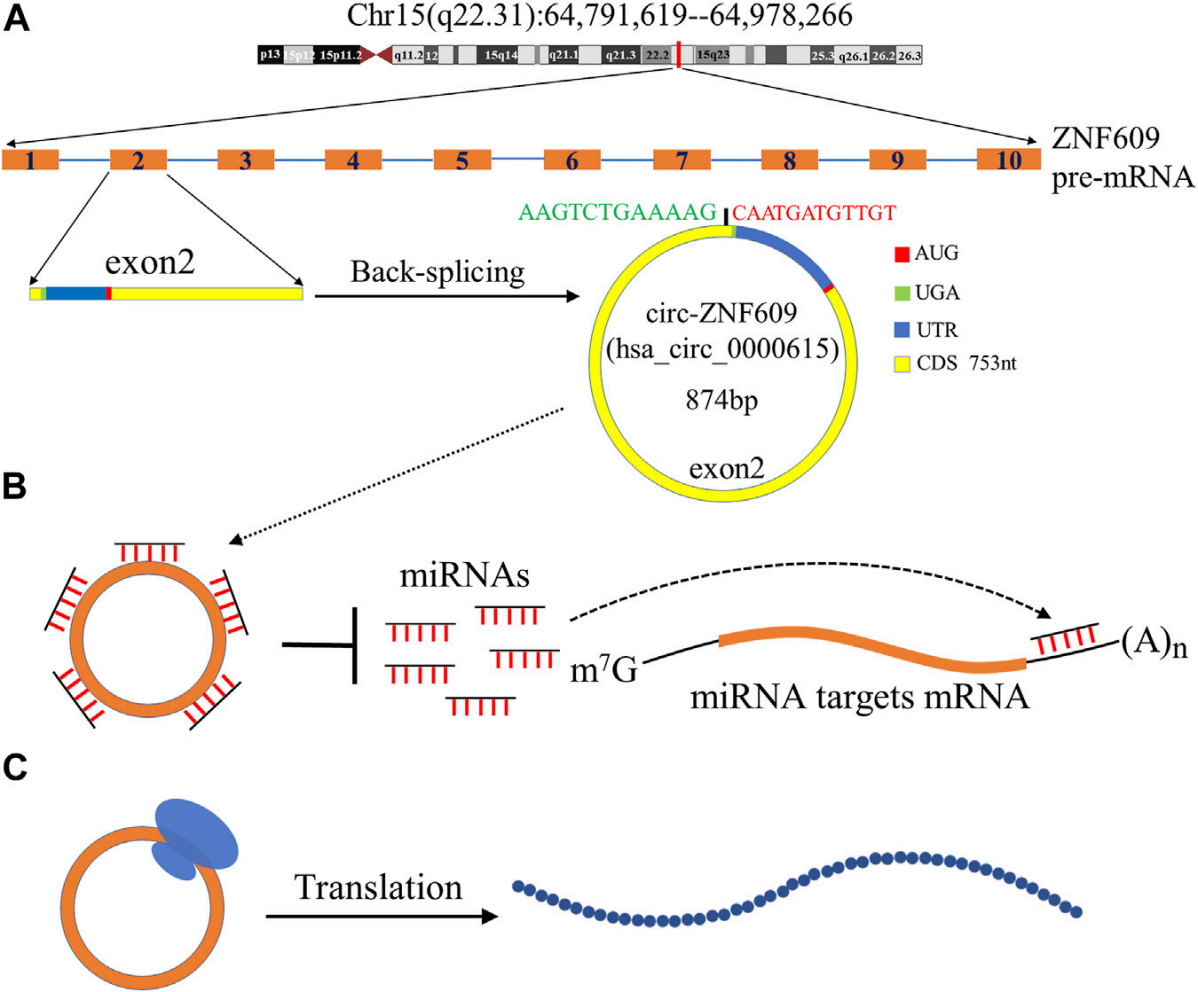
Biogenesis and biological functions of *circ-ZNF609*. **(A)** ZNF609 is located in chromosome 15q22.31 in humans; the red line indicates its approximate location. ZNF609 contains 10 exons, and the exon2 is spliced in reverse to form *circ-ZNF609*. The total length of *circ-ZNF609* is 874nt and contains a 753nt coding region, the promoter of *circ-ZNF609* is at position 128, and the terminator is at position 6. **(B)** *Circ-ZNF609* as a molecular sponge to adsorb microRNAs, reducing its binding to the 3′ UTR of the target mRNA, and regulate gene expression. **(C)** *Circ-ZNF609* can be translated into a protein.

*Circ-ZNF609* is a covalently closed circular RNA, which originates from the primary transcript of exon2 of ZNF609 located on chromosome 15q22.31. It is usually formed by back-splicing, a downstream splice-donor site is joined to an upstream splice-acceptor site, resulting in the loop structure of *circ-ZNF609* and containing a specific junction site. It contains 874 bp nucleotides (Legnini et al., 2017); as a kind of circRNA that could be translated, its coding sequence contains 753 bp nucleotides, the start codon is located at position 128, and the stop codon is located at position 6 (Figure 1). There is one of the RNA binding proteins that can regulate the biogenesis of *circ-ZNF609.* Liu et al. (Liu et al., 2021a) reported that the expression of *circ-ZNF609* could be upregulated by RNA binding protein fused in sarcoma (FUS), which could modulate the back-splicing reaction (Liu et al., 2021a). The FUS protein could induce the splicing and circularization of *circ-ZNF609* by binding to the upstream exon2 of ZNF609 pre-mRNA (Liu et al., 2021a).

Considering the loop structure of *circ-ZNF609* without a 5′ cap, its translation relies on a splicing-dependent/cap-independent manner (Legnini et al., 2017). However, Hung et al. (Ho-Xuan et al., 2020a) found that its translation may be originated from trans-splicing the byproducts of the overexpression of artificial circRNAs. They thought while performing functional studies of overexpression constructs of circRNA, it should be evaluated carefully (Ho-Xuan et al., 2020a). The primary biological function of *circ-ZNF609* was to act as a molecular sponge of endogenous microRNAs to sequester and inhibit the microRNA activity, which led to regulating the target gene expression (Chen, 2020; Xiao et al., 2020). As in hepatocellular carcinoma, *circ-ZNF609* inhibits *miR-15a-5p/15b-5p* expression and then elevates *GLI2* (a key protein molecule concerning the Hedgehog pathway) expression, activating the Hedgehog pathway to promote hepatocellular carcinoma (HCC) proliferation and metastasis (He et al., 2020).

### *Circ-ZNF609* in Multiple Human Cancers

**FIGURE 2:**
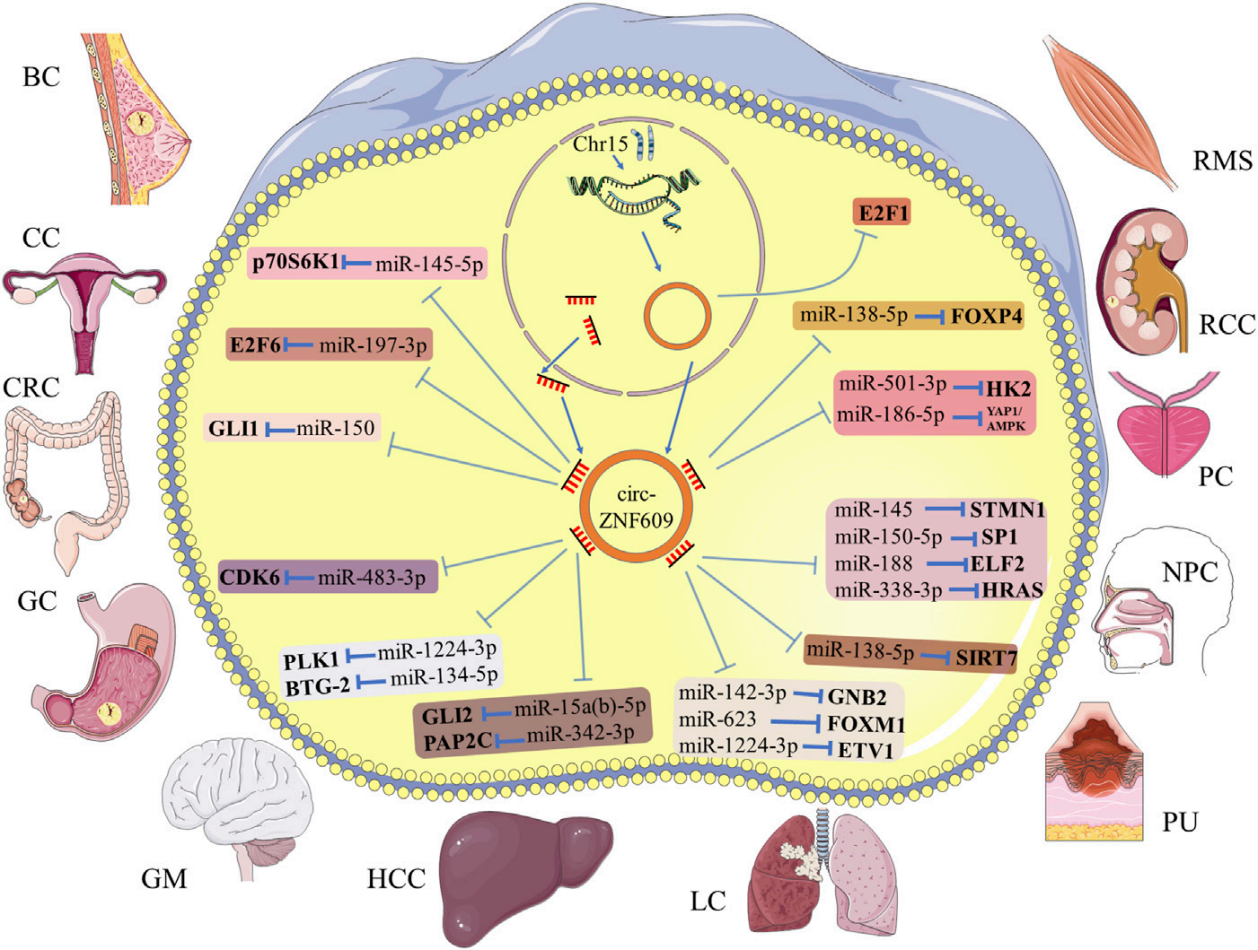
Schematic diagram for the molecular mechanism of *circ-ZNF609* in human cancers. *Circ-ZNF609* acts as a molecular sponge of specific miRNA to regulate the expression of targeted genes in multiple cancers. T-shaped bars represent inhibition.

The present studies suggested that the main function of *circ-ZNF609* is the posttranscriptional regulation, by acting as a molecular sponge of target microRNA (Figure 2; Table 1). As a carcinogen, *circ-ZNF609* was abnormally upregulated in tumor tissues and cell lines. It also promoted tumor proliferation, migration, invasion, and other malignant phenotypes. The following content describes the molecular mechanisms of *circ-ZNF609* in human cancers, in order by cancer names.

**TABLE 1:** Mechanism of *circ-ZNF609* in human cancers.

Cancers	Expression Change	Targeted miRNAs	Targeted Genes	Tumor’s progression	Clinicopathological Features	References
Breast cancer (BC)	Upregulated	miR-145-5p	p70S6K1	Promoting	Lymph node metastasis, advanced TNM stage, poor overall survival	Wang et al. (Wang et al., 2018a)
Cervical cancer (CC)	Upregulated	miR-197-3p	E2F6	Promoting	**—**	Gu et al*.* (Gu et al., 2021)
Colorectal cancer (CRC)	Upregulated	miR-150	GLI1	Promoting	Lymph node metastasis, Dukes stage	Wu et al. (Wu et al., 2018)
Gastric cancer (GC)	Upregulated	miR-483-3p	CDK6	Promoting	Advanced TNM stage, poor overall survival	Wu et al. (Wu et al., 2019)
Glioma (GM)	Upregulated	miR-1224-3p	PLK1	Promoting	Advanced clinical grade	Du et al*.* (Du et al., 2021)
		miR-134-5p	BTG-2	Promoting	**—**	Tong et al. (Tong et al., 2019)
Hepatocellular carcinoma (HCC)	Upregulated	miR-15a(b)-5p	GLI2	Promoting	**—**	He et al. (He et al., 2020)
		miR-342-3p	PAP2C	Promoting	Lymph node metastasis, advanced TNM stage, poor overall survival	Liao et al*.* (Liao et al., 2020)
Lung cancer (LC)	Upregulated	miR-142-3p	GNB2	Promoting	**—**	Liu et al*.* (Liu et al., 2021a)
		miR-623	FOXM1	Promoting	Lymph node metastasis, advanced TNM stage, poor overall survival	Wang et al. (Wang et al., 2021a)
		miR-1224-3p	ETV1	Promoting	**—**	Zuo et al. (Zuo et al., 2020)
Melanoma (MM)	Upregulated	miR-138-5p	SIRT7	Promoting	**—**	Liu et al. (Liu et al., 2021b)
Nasopharyngeal carcinoma (NPC)	Upregulated	miR-145	STMN1	Promoting	Lymph node metastasis, advanced clinical stage	Wang et al. (Wang et al., 2021b)
		miR-150-5p	SP1	Promoting	**—**	Zhu et al. (Zhu et al., 2019)
		miR-188	ELF2	Promoting	**—**	Li et al. (Li et al., 2020b)
		miR-338-3p	HRAS	Promoting	Poor overall survival	Liu et al. (Liu et al., 2021c)
Prostate cancer (PC)	Upregulated	miR-186-5p	YAP1/AMPK	Promoting	**—**	Jin et al. (Jin et al., 2019)
		miR-501-3p	HK2	Promoting	Advanced TNM stage, metastasis	Du et al. (Du et al., 2020)
Renal cell carcinoma (RCC)	Upregulated	miR-138-5p	FOXP4	Promoting	**—**	Xiong et al. (Xiong et al., 2019)
Rhabdomyosarcoma (RMS)	Upregulated	**—**	E2F1	Promoting	**—**	Rossi et al. (Rossi et al., 2019)

### Breast Cancer

Breast cancer is the most common cancer and the second leading cause of cancer lethality among women (DeSantis et al., 2017). In comparison with conventional surgery, neoadjuvant therapy has become a more widely used option, and novel targeted therapies play an important role in long-term disease control of metastatic breast cancer (Harbeck and Gnant, 2017). Being highly expressed in breast tumor tissues; *circ-ZNF609* leads to a poor outcome in the overall survival and is closely associated with lymph node metastasis and advanced TNM stage (Wang et al., 2018a). Wang et al. proved that *circ-ZNF609* knockdown inhibits the formation of malignant phenotypes of breast cancer cells and delays the tumor growth rate *in vivo*. It exerted biological function by sponging *miR145-5p*, which targeted oncogenic ribosomal protein S6 kinase, polypeptide 1 (*p70S6K1*), and promoted breast cancer progression (Wang et al., 2018a). They demonstrated that *circ-ZNF609* regulated the *miR-145-5p/p70S6K1* axis and could become a potential posttreatment prognostic biomarker in breast cancer.

### Cervical Cancer

Each year, more than 500,000 women are diagnosed with cervical cancer. Advances in radiotherapy technology have significantly reduced treatment-related toxicity (Cohen et al., 2019); however, the overall survival of metastatic cervical tumors is still poor. Gu et al. (Gu et al., 2021) found that *circ-ZNF609* was overexpressed in cervical cancer tissues and cell lines, and knockdown of *circ-ZNF609* suppresses the malignant phenotype of the tumor. Circ-ZNF609 acted as the sponge of *miR-197-3p*, directly upregulating the expression of E2F transcription factor 6 (*E2F6*). The overexpression of *E2F6* can partially reverse the inhibition of cell proliferation, migration, and invasion caused by *circ-ZNF609* depletion (Gu et al., 2021). Their research suggested that the *circ-ZNF609/miR-197-3p/E2F6* regulatory axis proposed a new insight into the progression of cervical cancer.

### Colorectal Cancer

Colorectal cancer is the third most commonly diagnosed cancer in males and the second in females (Torre et al., 2015). Exploring the potential diagnostic biomarkers of colorectal cancer is of great significance to contemporary medicine. Emerging evidence has shown the vital role of *circ-ZNF609* in colorectal cancer development and progression. The expression level of circ-ZNF609 was reported to be positively correlated with GLI family zinc finger 1 (*GLI1*) and negatively correlated with *miR-150*. In particular, it acted as a molecular sponge of *miR-150* to downregulate the expression of *GLI1* and then promoted colorectal cancer cell proliferation and migration (Wu et al., 2018). Later, Hung et al. (Ho-Xuan et al., 2020b) found that *circ-ZNF609* acted as an oncogene during colorectal cancer progression and metastasis. The overexpression of circ-ZNF609 leads to increased tumor growth, while knockdown led to contrasting effects in mouse xenograft models. However, Zhang et al. (Zhang et al., 2019a) reported discrepant results that *circ-ZNF609* is downregulated in colorectal cancer tissues and patient serum samples, and it also induced cell apoptosis *via* upregulating *p53*. To summarize, the specific role and regulating mechanism of *circ-ZNF609* in colorectal cancer are still unclear, requiring further elucidation.

### Gastric Cancer

Gastric cancer is the second leading cause of cancer-related deaths and the incidence ranks fourth worldwide, which mainly relies on pathological examination (Torre et al., 2015; Sitarz et al., 2018). Surgical resection and chemotherapy are the principal treatment approaches; however, lymph node and distant metastases during advanced stages limit the therapeutic effect (Sitarz et al., 2018). Therefore, seeking early biomarkers of gastric cancer makes all the difference between accurate diagnosis and treatment (Zhang and Zhang, 2017). Wu et al. (Wu et al., 2019) proved that *circ-ZNF609* is overexpressed in cancer tissues and cell lines of gastric cancer patients, and it was positively correlated with a higher TNM stage and a lower 5-year survival rate. It acted as a sponge of *miR-483-3p*, upregulated the expression of cell-promoting factor cyclin-dependent kinase 6 (*CDK6*), and promoted the proliferation and migration of gastric cancer cells through the *circ-ZNF609/miR-483-3p/CDK6* axis (Wu et al., 2019). In interest, Liu et al. (Liu et al., 2019) had discovered different mechanisms of *circ-ZNF609* in gastric cancer, through binding to *miR-145-5p* and negatively regulating its expression. Knockdown of *circ-ZNF609* inhibited cell proliferation and induced apoptosis, which could be partially reversed by *miR-145-5p* overexpression (Liu et al., 2019).

### Glioma

Glioma is a primary brain tumor that is highly metastatic and aggressive (Weller et al., 2015; Chen et al., 2017). It is of great significance to determine the potential molecular mechanism of *circ-ZNF609* in glioma. Du et al. (Du et al., 2021) proved that *circ-ZNF609* was overexpressed in glioma tissues and cell lines and was significantly overexpressed in high-grade glioma than in low-grade glioma. Silencing *circ-ZNF609* could inhibit the proliferation and migration of glioma cells. In routine, it promoted the expression of polo-like kinase-1 (*PLK1*) by competitively binding to *miR-1224-3p*, and *circ-ZNF609* also promoted tumor growth *in vivo* (Du et al., 2021). Meanwhile, Tong et al. (Tong et al., 2019) found that *miR-134-5p* inhibited the expression of BTG antiproliferation factor 2 (*BTG-2*) and inhibited the proliferation and migration of glioma. *Circ-ZNF609* positively regulated the expression of *BTG-2* through competitively binding to *miR-134-5p*, leading to the proliferation and migration of glioma. It proposed a novel mechanism of *circ-ZNF609* in regulating the progression of glioma (Tong et al., 2019).

### Hepatocellular Carcinoma

Liver cancer is the fourth leading cause of cancer deaths worldwide (Villanueva, 2019). Existing studies have demonstrated that circRNAs could promote the progress of HCC by regulating microRNAs (Zhang and Wang, 2021). *Circ-ZNF609* was highly expressed in HCC tissues and cell lines. Knockdown of *circ-ZNF609* could inhibit the proliferation, migration, and invasion of HCC and promote apoptosis (He et al., 2020). Adding SAG, an agonist of the Hedgehog signal pathway, could restore the phenotype caused by *circ-ZNF609* knockdown (He et al., 2020). Bioinformatics analysis and experiments validated that *circ-ZNF609* regulated the expressions of *miR-15a-5p/15b-5p* and GLI family zinc finger 2 (*GLI2*) and promoted the malignant phenotype of HCC through the Hedgehog pathway (He et al., 2020). Liao et al. (Liao et al., 2020) also proved that silencing *circ-ZNF609* could inhibit the proliferation of HCC and upregulate the expression of *RAP2C* (member of the RAS oncogene family) by acting as a sponge of *miR-342-3p*. The experiments *in vivo* showed that *circ-ZNF609* facilitated tumor growths, confirming the findings *in vitro*.^49^

### Lung Cancer

Non-small cell lung cancer (NSCLC) is the most common type of lung cancer (Devesa et al., 2005), and circRNAs play a significant role in its pathogenesis and progression (Di et al., 2019; Huang et al., 2019). Fork head box protein M1 (*FOXM1*) is overexpressed in various cancers, which is a necessary transcription factor for cell proliferation (Liao et al., 2018). In NSCLC, it was targeted by miR-623 and *circ-ZNF609*. Knocking down *circ-ZNF609* inhibited cell viability, migration, and invasion and promoted apoptosis, and knocking down *miR-623* or overexpressed *FOXM1* could weaken these effects (Wang et al., 2021a). Lung adenocarcinoma (LUAD) is one of the histological subtypes of lung cancer with a poor prognosis (Devesa et al., 2005); *circ-ZNF609* was overexpressed in LUAD, acting as a sponge of *miR-1224-3p* to promote the cell proliferation of LUAD, which negatively regulated the expression of ETS variant transcription factor 1 (*ETV1*). They verified that *circ-ZNF609* promoted LUAD proliferation through the *miR-1224-3p/ETV1* axis (Zuo et al., 2020). In lung cancer, it was also found that FUS RNA binding protein could bind to the intron1 region of pre-mRNA of ZNF609, but not to exon1 and exon2. The specific binding may regulate the back-splicing of exon2, leading to upregulation of *circ-ZNF609* and promoting the malignant progression of lung cancer through the *miR-142-3p/GNB2* axis (Liu et al., 2021a). These studies provided different insights for understanding the value of *circ-ZNF609* in different histological subtypes of lung cancer, indicating that the pathogenic mechanism of *circ-ZNF609* in lung cancer was tissue specific.

### Melanoma

Melanoma is a prevalent malignant skin cancer. Its incidence and mortality rates vary greatly worldwide. Once melanoma spreads, it will quickly become life-threatening (Schadendorf et al., 2018). As stated, *circ-ZNF609*-mediated DNA damage plays an important role in the development of melanoma. Knocking down *circ-ZNF609* could inhibit the proliferation, migration, and invasion of melanoma cell lines, reduce cell survival rate, and promote apoptosis (Liu et al., 2021b). Comet assays showed that the tail length was elevated and the expression level of γH2AX variant histone (*γH2AX*) was increased after *circ-ZNF609* depletion, suggesting that *circ-ZNF609* inhibited the DNA damage in melanoma (Liu et al., 2021b). *Circ-ZNF609* repressed DNA oxidative damage by acting as a sponge of *miR-138-5p*, which induced DNA oxidative damage by targeting sirtuin 7 (*SIRT7*). Adding *miR-138-5p* inhibitor or overexpression of *SIRT7* partially reversed the DNA damage phenotype caused by *circ-ZNF609* depletion, and *circ-ZNF609* depletion reduced the tumor size, tumor volume, and tumor weight through the *miR-138-5p/SIRT7* axis *in vivo* (Liu et al., 2021b). This study provided a new mechanism for the pathogenesis of DNA damage in melanoma, suggesting that circRNA-mediated DNA oxidative damage may be a valuable direction for melanoma biogenesis.

### Nasopharyngeal Carcinoma

Nasopharyngeal carcinoma (NPC) is a common malignant tumor of the head and neck, and chemotherapy is an effective treatment method (Chen et al., 2019). However, since it is prone to lymph node metastasis and the degree of malignancy is high (Chua et al., 2016), it is of great significance to study the mechanism of occurrence and development. Pathological angiogenesis is a hallmark of cancer progression (Carmeliet and Jain, 2000), which is an important cause of the metastasis of NPC (Bao et al., 2018). The expression of vascular endothelial growth factor (VEGF) after the knockdown of *circ-ZNF609* in NPC cells was downregulated. The supernatant was added to treat human umbilical vein endothelial cells (HUVEC), and the proteins of VEGF receptor-1 and VEGF receptor-2 in HUVECs are reduced. It could be observed that the total tube length was shortened, and the nodules were reduced when knocking down *circ-ZNF609* in HUVEC. These proved that the angiogenesis was reduced after knocking down *circ-ZNF609*. It negatively regulated the expression of *miR-145* and upregulated stathmin 1 (*STMN1*) to promote the proliferation, migration, and angiogenesis of NPC, forming a new regulatory mechanism for the pathological angiogenesis of NPC (Wang et al., 2021b). Zhu et al. (Zhu et al., 2019) found that *circ-ZNF609* promoted the growth and metastasis of NPC and exerted carcinogenic influence by competing with *miR-150-5p* which degraded *Sp1* expression. Liu et al. (Liu et al., 2021c) also proposed that *circ-ZNF609* was highly expressed in NPC tissues and cell lines, by binding to *miR-338-3p* to negatively regulate its expression, upregulating histidyl-tRNA synthetase (HARS) that promoted the proliferation, migration, invasion, and glycolysis of NPC, and xenograft experiments proved the result *in vitro*. The results of Li et al. (Li et al., 2020b) also showed that *circ-ZNF609* was overexpressed in NPC tissues and cell lines, knocking down it inhibited NPC cell proliferation and cell cycle transition, as well as accelerated apoptosis, and the carcinogenic effect was achieved through the *circ-ZNF609/miR-188/ELF2* axis. Their studies had shown that *circ-ZNF609* was overexpressed in NPC, as a molecular sponge of related microRNA and upregulated the expression of the target gene, achieving carcinogenic effects, and these suggest that *circ-ZNF609* may be a new therapeutic target for NPC. The molecular mechanism of *circ-ZNF609* in NPC had been inconsistently reached by different research teams, which may be caused by differences in patient samples. The mechanism of *circ-ZNF609* in NPC required a more rigorous study to reveal a clear conclusion.

### Prostate Cancer

Prostate cancer is a common malignant tumor of the urinary system (Torre et al., 2015), and radiotherapy is the main treatment modality. However, the metastasis of advanced patients limits the application of radiotherapy (Mohler et al., 2010). It is of great significance to understand the mechanisms of radiological resistance. Even in the presence of oxygen and fully functional mitochondria, tumor cells increased glucose uptake and fermentation of glucose to lactate, and the process is called the Warburg effect. It is characterized by changes in glycolysis and metabolism, which can promote tumor metastasis (Liberti and Locasale, 2016). *Circ-ZNF609* was highly expressed in prostate cancer tissues and cells, and silencing it could repress cell viability, inhibit cell migration and invasion, and induce cell apoptosis (Du et al., 2020). *Circ-ZNF609* silencing decreased the glucose uptake and lactate product of tumor cells, and overexpression of *circ-ZNF609* could increase the radioresistance of cells. However, the radioresistance was significantly inhibited by the addition of glycolysis inhibitor 2-deoxy-D-glucose (2-DG), suggesting that *circ-ZNF609* promoted glycolysis to improve the radioresistance of cells (Du et al., 2020). Circ-ZNF609 acted as a molecular sponge of *miR-501-3p*, and 2-DG could significantly inhibit the promotion of glycolysis by anti-*miR-501-3p*. The *circ-ZNF609/miR-501-3p* axis was targeted to upregulate the expression of hexokinase 2 (*HK2*), a key enzyme of glycolysis, and then improved the radioresistance of tumor cells both *in vitro* and *in vivo* (Du et al., 2020). Jin et al. (Jin et al., 2019) proposed that silencing *circ-ZNF609* could restrain Yes1-associated transcriptional regulator (*YAP1*) and AMP-activated protein kinase (*AMPK*) signaling pathways by upregulating *miR-186-5p*, thereby inhibiting cell proliferation, migration, and invasion, and inducing apoptosis. In conclusion, *circ-ZNF609* could promote prostate cancer progression through multiple mechanisms, including regulated glycolysis and metabolism, promoting radioresistance and activating signaling pathways.

### Renal Cell Carcinoma

Renal cell carcinoma (RCC) is a common tumor of the urinary system (Rini et al., 2009). Some ncRNAs have been proved to be involved in the biological process of kidney cancer, providing molecular targets for the treatment (Li et al., 2017; Wang et al., 2017; Shelar et al., 2018). Xiong et al. (Xiong et al., 2019) proved that *circ-ZNF609* represented a circular structure that was resistant to the digestion of RNase R. It was also overexpressed in renal cancer cell lines than in renal epithelial cells. By targeted binding to *miR-138-5p*, *circ-ZNF609* upregulated the expression of the transcription factor forkhead box P4 (*FOXP4*) and promoted the proliferation, migration, and invasion of renal cancer cells. Knocking down of *circ-ZNF609* inhibited the malignant phenotype in RCC (Xiong et al., 2019).

### Rhabdomyosarcoma

RMS is a pediatric skeletal muscle malignancy that accounts for roughly 5% of all pediatric tumors (Egas-Bejar and Huh, 2014). Rhabdomyosarcoma in children is usually divided into two main histological subtypes, the embryonal rhabdomyosarcoma (ERMS) and the alveolar rhabdomyosarcoma (ARMS), and the latter has a generally worse prognosis (Egas-Bejar and Huh, 2014; Sun et al., 2015). Rossi et al. (Rossi et al., 2019) reported that *circ-ZNF609* was upregulated in biopsies from ERMS and ARMS. *Circ-ZNF609* knockdown induced a significant decrease in the p-*Akt* protein level, which modulated cell proliferation-related pathways and an alteration of the p-*Rb/Rb* ratio in an ERMS-derived cell line. The hypophosphorylated Rb protein could bind E2F transcription factor 1 (*E2F1*) to reduce the activation of S-phase transcription factors such as *TCF19* and *MCMs*, which caused a specific block of ERMS from the G1 to the S phase. Differently to ERMS, in the ARMS-derived cells, due to the lower *p53* that was involved in cell cycle arrest, ARMS does not undergo G1-S arrested after the *circ-ZNF609* knockdown, which means that *circ-ZNF609* knockdown was not enough to significantly inhibit ARMS cell proliferation (Rossi et al., 2019).

### *Circ-ZNF609* in Other Human Diseases

**FIGURE 3:**
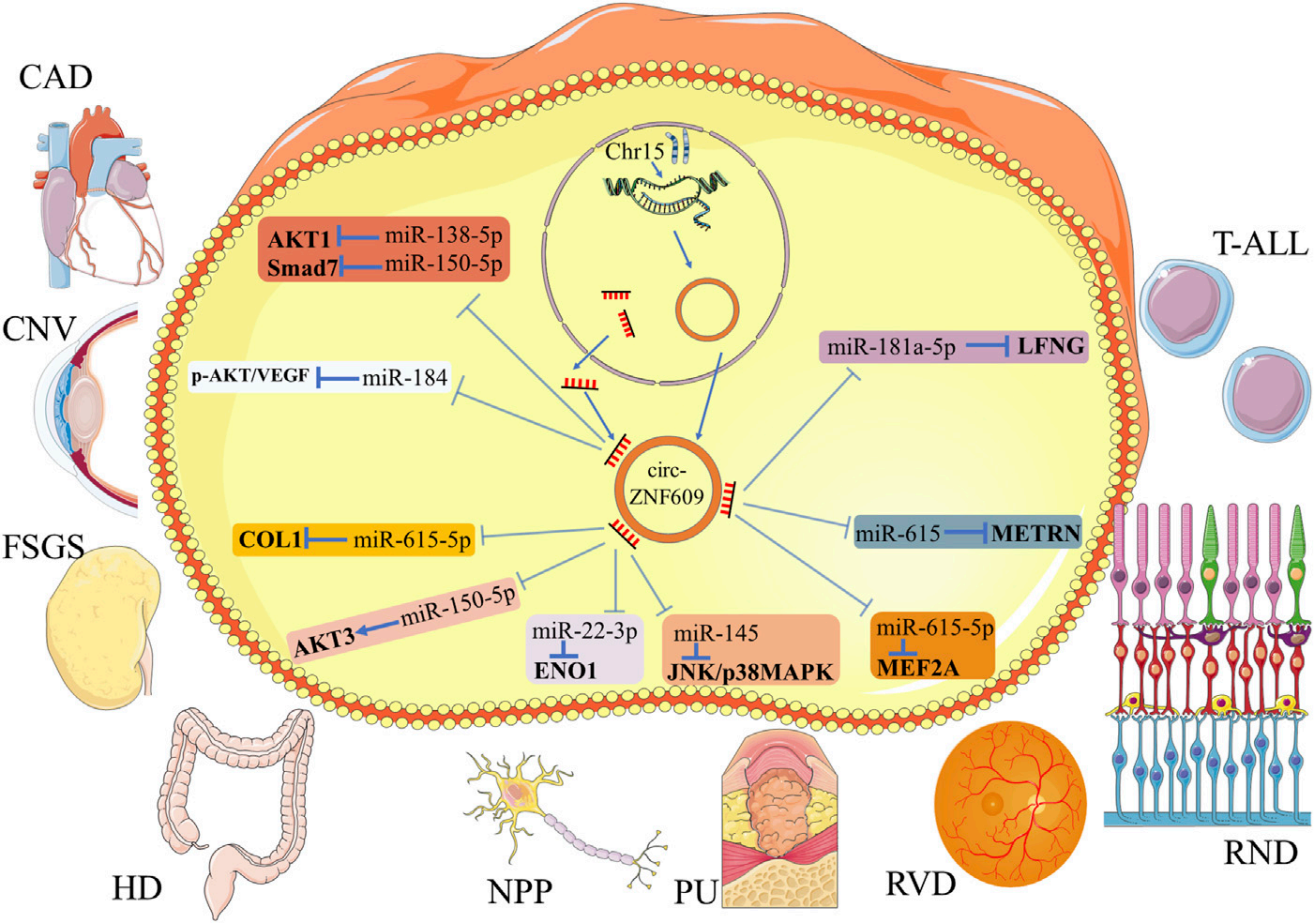
Schematic diagram for the molecular mechanism of *circ-ZNF609* in nontumor diseases. *Circ-ZNF609* acts as a molecular sponge of specific miRNA to regulate the expression of targeted genes in nontumor diseases. T-shaped bars represent inhibition, and arrows represent promotion.

Similar to the function in human tumors, *circ-ZNF609* also acted as a sponge of microRNAs in nontumor diseases (Figure 3; Table 2). In nontumor diseases, the expression level of *circ-ZNF609* had not been verified in the human tissues. On accounting for cell lines and animal models, researchers found that *circ-ZNF609* promoted cell proliferation and induced poor phenotypes in most nontumorous diseases. However, in coronary heart disease and Hirschsprung’s disease, it was downregulated, representing an opposite function as a protective regulator. Therefore, we summarized the role of *circ-ZNF609* in nontumorous diseases but not only in human cancers here.

**TABLE 2:** Mechanism of *circ-ZNF609* in other human diseases.

Diseases	Expression Change	Targeted miRNAs	Targeted genes	Diseases Progression	References
Coronary artery disease (CAD)	Downregulated	miR-138-5p^a^	AKT1^a^	Inhibiting	Liang et al. (Liang et al., 2020)
		miR-150-5p^a^	Smad7^a^		
Corneal neovascularization (CNV)	Upregulated	miR-184	p-AKT/VEGF	Promoting	Wu et al. (Wu et al., 2020)
Focal segmental glomerulosclerosis (FSGS)	Upregulated	miR-615-5p	COL1	Promoting	Cui et al. (Cui et al., 2020)
Hirschsprung’s disease (HD)	Downregulated	miR-150-5p	AKT3	Inhibiting	Peng et al. (Peng et al., 2017)
Neuropathic pain (NPP)	Upregulated	miR-22-3p	ENO1	Promoting	Li et al. (Li et al., 2020c)
Pressure ulcer (PU)	Upregulated	miR-145	JNK/p38MAPK	Promoting	Ge and Gao (Ge and Gao, 2020)
Retinal neurodegeneration (RND)	Upregulated	miR-615	METRN	Promoting	Wang et al. (Wang et al., 2018b)
Retinal vascular dysfunction (RVD)	Upregulated	miR-615-5p	MEF2A	Promoting	Liu et al. (Liu et al., 2017)
T-cell acute lymphoblastic leukemia (T-ALL)	Upregulated	miR-181a-5p	LFNG	Promoting	Buratin et al. (Buratin et al., 2020)

a Based on bioinformatics prediction and literature reports.

### Coronary Artery Disease

Coronary artery disease (CAD) is the major cause of mortality globally (Dagenais et al., 2020), and the inflammatory response theory has been widely recognized in its pathogenic mechanism (Hansson, 2005). It has been reported that circRNAs were involved in several cardiovascular pathological processes (Wang et al., 2016; Shen et al., 2019). Compared with normal populations, the expression of *circ-ZNF609* was downregulated in CAD patients. Logistic analysis suggested that a low *circ-ZNF609* level was an independent risk factor for CAD. Overexpression of *circ-ZNF609* in cells would cause the decrease of *IL-6* and *TNF-α* and an increase in *IL-10* expressions, suggesting its antiinflammatory effects, and could alleviate the development of CAD (Liang et al., 2020). Based on the bioinformatics prediction and the literature reports, researchers speculated that *circ-ZNF609* exerted a protective function in CAD by sponging microRNA and regulated the *miR-138-5p/AKT1* or *miR-150-5p/Smad7* axis to interrupter inflammation pathways.

### Corneal Neovascularization

The cornea lacks blood vessels to ensure that light passes through the lens, and pathological corneal neovascularization derived from the corneal limbus which is filled with blood vessels can affect the function of the transparent cornea and threaten vision (Feizi et al., 2017; Mobaraki et al., 2019). The study of corneal neovascularization by Wu et al. (Wu et al., 2020) suggested that in the rat corneal suture model, the overexpression of *circ-ZNF609* and a decrease of *miR-184* were observed in the corneal epithelia of rats after corneal suture surgery, and *circ-ZNF609* acted as a sponge of *miR-184* to regulate the *AKT/β-catenin/VEGF* signaling pathway and then promoted cell proliferation, migration *in vitro*, and angiogenesis *in vivo*.^84^ Their study proved that inhibiting *circ-ZNF609* may be a new therapeutic method for the treatment of pathological corneal neovascularization. The role of *circ-ZNF609* in rat corneal neovascularization was different from that of human tumors; although there was no more cell line model to validate the finding, it could be validated in more animal models to ensure the conclusion.

### Focal Segmental Glomerulosclerosis

Focal segmental glomerulosclerosis (FSGS) is the main cause of kidney disease worldwide, and it is a complex syndrome that arises after podocyte injury in general (Rosenberg and Kopp, 2017). In the mice model of FSGS, the expression of *circ-ZNF609* was increased in biopsies compared to normal control mice. The expression of *circ-ZNF609* was positively correlated with the degree of podocyte destruction and renal fibrosis, but *miR-615-5p* was negatively correlated with *circ-ZNF609* (Cui et al., 2020). In mechanism, it acted as a molecular sponge of *miR-615-5p* to downregulate the expression of podocyte biomarkers *WT1* and upregulate fibrotic proteins including *COL1*, promoting the progression of FSGS (Cui et al., 2020). The expression of *circ-ZNF609* in the kidney was limited not only to RCC but also to FSGS, indicating that the scope of research could be extended to other nontumor sites. This study suggested that *circ-ZNF609* might be a potential biomarker for the diagnosis of kidney disease.

### Hirschsprung’s Disease

Hirschsprung’s disease (HSCR) is caused by a lack of enteric nerve cells in the variable part of the distal intestine, and infants with related genetic changes usually develop intestinal obstruction a few days after birth (Kenny et al., 2010). The expression of *circ-ZNF609* was downregulated in HSCR compared with normal colon tissues and inhibited the proliferation and migration of HSCR cells. In mechanism, it downregulated the expression of AKT serine/threonine kinase 3 (*AKT3*) through acting as a sponge of *miR-150-5p* and promoted disease progression (Peng et al., 2017). However, Rossi et al. (Rossi et al., 2019) found that the expressions of *miR-150-5p* and *AKT3* were not affected by the downregulation of *circ-ZNF609*; therefore, the mechanism of *circ-ZNF609-*regulated HSCR progression needs further study.

### Neuropathic Pain

The widely accepted definition of neuropathic pain is the pain caused by a lesion or disease of the somatosensory system (Colloca et al., 2017). Due to the aging of the global population, the increasing incidence of cancer, and the consequences of chemotherapy, neuropathic pain may become more common (Colloca et al., 2017), and therefore finding its therapeutic targets has important clinical significance. The expression level of *miR-22-3p* was reduced in rat models with chronic constrictive injury and was involved in the progression of neuropathic pain, and *miR-22-3p* downregulation promoted neuropathic pain by targeting enolase 1 (*ENO1*) to regulate the expression of inflammatory factors (Li et al., 2020c). Li et al. (Li et al., 2020c) found that *circ-ZNF609* regulated the expression of inflammatory factors *TNF-α*, *IL-1*, and *IL-6* to promote neuropathic pain progression through the *miR-22-3p/ENO1* axis (Li et al., 2020c). Although it was inappropriate to quantify the neuropathic pain phenotype with the level of inflammatory factors, this paper provided a new molecular mechanism for *circ-ZNF609* in the regulation of inflammatory factors.

### Pressure Ulcers

Pressure ulcers (PU) mostly occur in paralyzed and bedridden patients and are localized injuries to the skin and/or underlying tissues, usually over a bony prominence as a result of pressure combined with friction (Agrawal and Chauhan, 2012). PU are usually accompanied by skin oxidative damage, and drugs with antioxidant function are considered for treatment (Liu et al., 2018; Zhang et al., 2019b). In the PU model of HaCaT cells treated with H_2_O_2_, the expression of *circ-ZNF609* was promoted in the model, and silencing of the expression of *circ-ZNF609* alleviated oxidative stress damage including the viability loss, apoptosis, and ROS generation of HaCaT cells, through inhibiting the *JNK* and *p38MAPK* signaling pathways via acting as the sponge of *miR-145* (Ge and Gao, 2020). The study in the model of PU provided new evidence for *circ-ZNF609* in oxidative stress damage.

### Retinal Neurodegeneration

Glaucoma is mainly manifested by visual field loss and irreversible blindness caused by progressively retinal neurodegenerative diseases, and the death of retinal ganglion cells (RGC) and high intraocular pressure are pathophysiological characteristics (Almasieh et al., 2012; Danesh-Meyer and Levin, 2015). In the rat model, *circ-ZNF609* was significantly upregulated in the degeneration of the optic nerve induced by high intraocular pressure, and silencing it could inhibit the proliferation of RGC and optic nerve damage caused by high intraocular pressure (Wang et al., 2018b). Their previous studies suggested that *circ-ZNF609* acted as a sponge of *miR-615* to promote the proliferation of vascular endothelial cells. *Circ-ZNF609* in RGC also acted as a sponge of *miR-615* and then upregulated mentoring glial cell differentiation regulator (*METRN*) expression, promoting cell proliferation (Wang et al., 2018b). This study of *circ-ZNF609* provided new insight into circRNAs in the growth of nerves, and it reflected the breadth of the role of circRNAs.

### Retinal Vascular Dysfunction

Vascular dysfunction is a hallmark of pathological angiogenesis and contributes to the progression of various diseases (Puro et al., 2016), the expression of circRNAs is dysregulated in cardiovascular disease that is accompanied by vascular dysfunction, and endothelial cell regulation plays an important role in it (Eelen et al., 2015; Aufiero et al., 2019). The retinal vascular system can be observed by noninvasive means, which can be used to investigate the mechanism of vascular dysfunction (Flammer et al., 2013). *Circ-ZNF609* promoted pathological angiogenesis and made endothelial cells more susceptible to oxidative stress and hypoxia (Liu et al., 2017; Wang et al., 2021b). Knocking down *circ-ZNF609* in the mouse model of oxygen-induced retinopathy did not affect the development of normal retinal vascular, while it reduced avascular area and reduced pathological retinal angiogenesis (Liu et al., 2017). The study revealed the mechanism of action for the *circ-ZNF609/miR-615-5p/MEF2A* axis in the mediation of vascular endothelial dysfunction, and since pathological angiogenesis is a hallmark of tumors, this study also verified that *circ-ZNF609* can cause tumors.

### T-Cell Acute Lymphoblastic Leukemia

The expression level of circRNAs differs among normal blood cell types, but the expression in T-ALL is still unclear (Nicolet et al., 2018). RNA sequencing data from 25 T-ALL patients were analyzed and most circRNAs were found to be downregulated in expression in malignant T-ALL (Buratin et al., 2020). In particular, *circ-ZNF609* was overexpressed in immature T-ALL, knocking down *circ-ZNF609*-inhibited cell proliferation and survival compared with normal control (Buratin et al., 2020). Bioinformatics analysis suggested that *circ-ZNF609* was bound to *miR-181a-5p* in immature T-ALL, which by targeting O-fucosylpeptide 3-beta-N-acetylglucosaminyltransferase (*LFNG*) to promote leukemogenic potential through the Notch1 signaling pathway in T-ALL (Buratin et al., 2020). With only the predicted results of the bioinformatics analysis available here, researchers could verify the molecular mechanism of *circ-ZNF609* in T-ALL through more cell experiments and animal experiments.

## Conclusion and Future Prospects

The present review described the basic biological functions of *circ-ZNF609* and systematically concluded its differential expression and its underlying molecular mechanism in human diseases. We found that the expression of *circ-ZNF609* in various cancer tissues was higher than in adjacent normal tissues and dysregulation in nontumor diseases. When *circ-ZNF609* is overexpressed in tumor tissues, it could result in poor overall survival, and it positively correlated with lymph node metastasis and advanced TNM or clinical stage. Relevant research on *circ-ZNF609* helped us understand the pathogenesis of many diseases, and it was proved that *circ-ZNF609* might be an effective and promising biomarker for diagnosis.

CircRNAs not only exist in human tissues and blood cells but are also differentially expressed in the serum and exosomes, playing an important role in disease progression (Devaux, 2017; Vea et al., 2018; Kristensen et al., 2019; Wang et al., 2019). Therefore, researchers can use the circRNAs in the patient’s body fluid as a noninvasive molecular marker. Emerging circRNAs have become potential therapeutic targets for human diseases, and *circ-ZNF609* is one of them. In particular, *circ-ZNF609* is an RNA that can be translated into a protein, giving it a broader role. Nowadays, the COVID-19 epidemic is serious, and the mRNA-based vaccine still has its limitations (Alameh et al., 2020; Corbett et al., 2020). The loop structure of *circ-ZNF609* prevents its degradation and confers stronger stability to it compared with linear mRNA (Xiao and Wilusz, 2019). It is a promising research direction to develop a circRNA-based vaccine, by integrating an antigen-encoding sequence of COVID-19 into *circ-ZNF609*. The internal ribosomal entry site of *circ-ZNF609* confers the translational function*,* making it possible to express the antigen of COVID-19. Qu et al. (Qu et al., 2022) reported a circRNA vaccine that elicited potent neutralizing antibodies and T-cell responses in an animal model (Qu et al., 2022; Szabó et al., 2022). The above studies indicated that *circ-ZNF609* might become an effective and safe molecular platform against the epidemic.

In human diseases, it exerts functions through the *circ-ZNF609*-miRNA-mRNA network, and *circ-ZNF609* knockdown or overexpression of miRNA will inhibit the malignant phenotype of cancers. Therefore, knocking down *circ-ZNF609* by precise RNA interference (RNAi) or knocking out by CRISPR/Cas9-mediated circRNA knockout (Yang et al., 2018), developing miRNA inhibitors, could serve as potential therapeutic strategies for treatments of multiple human diseases. In comparison with Qian et al. (Qian et al., 2021), we comprehensively discussed the underlying molecular mechanism of *circ-ZNF609* in multiple human diseases and expanded the types of the disease in detail. In addition, we discussed future research directions in *circ-ZNF609*-dysregulated diseases. The possible function of *circ-ZNF609* in the prevention of the COVID-19 epidemic was also explored. We hope this study could help reveal the far-reaching clinical significance of *circ-ZNF609*.
